# Neonatal Clonazepam Administration Induces Long-Lasting Changes in Glutamate Receptors

**DOI:** 10.3389/fnmol.2018.00382

**Published:** 2018-10-11

**Authors:** Hana Kubová, Zdenka Bendová, Simona Moravcová, Dominika Pačesová, Luisa Lilia Rocha, Pavel Mareš

**Affiliations:** ^1^Institute of Physiology, Academy of Sciences of the Czech Republic, Prague, Czechia; ^2^Faculty of Science, Charles University, Prague, Czechia; ^3^National Institute of Mental Health, Klecany, Czechia; ^4^Pharmacobiology Department, Center of Research and Advanced Studies, Mexico City, Mexico

**Keywords:** neonatal rat, benzodiazepine, clonazepam, NMDA receptor subunits, AMPA receptor subunits, mRNA expression, NMDA receptor autoradiography, [^3^H] MK-801

## Abstract

γ-aminobutyric acid (GABA) pathways play an important role in neuronal circuitry formation during early postnatal development. Our previous studies revealed an increased risk for adverse neurodevelopmental consequences in animals exposed to benzodiazepines, which enhance GABA inhibition via GABA_A_ receptors. We reported that administration of the benzodiazepine clonazepam (CZP) during postnatal days 7–11 resulted in permanent behavioral alterations. However, the mechanisms underlying these changes are unknown. We hypothesized that early CZP exposure modifies development of glutamatergic receptors and their composition due to the tight developmental link between GABAergic functions and maturation of glutamatergic signaling. These changes may alter excitatory synapses, as well as neuronal connectivity and function of the neural network. We used quantitative real-time PCR and quantitative autoradiography to examine changes in NMDA and AMPA receptor composition and binding in response to CZP (1 mg/kg/day) administration for five consecutive days, beginning on P7. Brains were collected 48 h, 1 week, or 60 days after treatment cessation, and mRNA subunit expression was assessed in the hippocampus and sensorimotor cortex. A separate group of animals was used to determine binding to NMDA in different brain regions. Patterns of CZP-induced alterations in subunit mRNA expression were dependent on brain structure, interval after CZP cessation, and receptor subunit type. In the hippocampus, upregulation of GluN1, GluN3, and GluR2 subunit mRNA was observed at the 48-h interval, and GluN2A and GluR1 mRNA expression levels were higher 1 week after CZP cessation compared to controls, while GluN2B was downregulated. CZP exposure increased GluN3 and GluR2 subunit mRNA expression levels in the sensorimotor cortex 48 h after treatment cessation. GluA3 was higher 1 week after the CZP exposure, and GluN2A and GluA4 mRNA were significantly upregulated 2 months later. Expression of other subunits was not significantly different from that of the controls. NMDA receptor binding increased 1 week after the end of exposure in most hippocampal and cortical areas, including the sensorimotor cortex at the 48-h interval. CZP exposure decreased NMDA receptor binding in most evaluated hippocampal and cortical areas 2 months after the end of administration. Overall, early CZP exposure likely results in long-term glutamatergic receptor modulation that may affect synaptic development and function, potentially causing behavioral impairment.

## Introduction

Since the introduction of benzodiazepines (BZDs) into clinical practice, these drugs have been among the most frequently used, and their stable efficacy throughout development has been documented in both clinical and preclinical studies (for rev., Farrell, 1986; Kubová et al., 1993). BZDs are used for a wide variety of medical indications in all patient age groups, including newborns because these agents exhibit distinct anticonvulsant, sedative, anxiolytic, hypnotic, and myorelaxant effects (Rennie and Boylan, 2003). The effects of early BZD exposure on brain development are of great concern due to use of these agents in pediatric patients. Studies in rodents demonstrated that BZD exposure during brain development resulted in persistent modification of brain function, behavioral alterations, and cognitive deficits (for review Gai and Grimm, 1982; Tucker, 1985; Kellogg, 1988). Enduring behavioral, biochemical, and molecular effects in response to early BZD exposure also occurs when these drugs are administered after neuronal differentiation but before complete maturation of the central nervous system, that is, during the first 3 weeks of life in rats (Avishai-Eliner et al., 2002).

The effects of BZDs are mediated via interaction with the BZD receptor-binding site, which modulates the efficacy of the major inhibitory neurotransmitter, *γ*-aminobutyric acid (GABA), on GABA_A_ receptors. Long lasting increases of GABA-mediated inhibition during chronic BZD administration triggers adaptive processes that involve GABAergic and glutamatergic systems. The impact of BZD exposure on the glutamatergic system has been extensively studied in mature animals, and transient changes associated with withdrawal phenomena and tolerance development have been reported (for rev. Allison and Pratt, 2003; Uusi-Oukari and Korpi, 2010).

Development of the GABAergic system is tightly associated with NMDA and AMPA receptor development. GABA serves as a major inhibitory neurotransmitter in the adult brain, but it plays multiple roles during early development. GABA operates as an excitatory neurotransmitter in neonatal neurons, and its depolarizing effects gradually change to hyperpolarizing effects with maturation of Cl^-^ homeostasis systems (Ben-Ari et al., 1997). However, previous studies suggest that GABA exerts dual effects on immature neurons. In addition to its excitatory depolarizing effects, GABA also exerts inhibitory shunting effects that are observed in mature neurons (Staley and Mody, 1992; Chen et al., 1996).

The switch in GABAergic effects from depolarizing to hyperpolarizing parallels upregulation of NMDA and AMPA receptor expression that occurs during the first postnatal week (Ben-Ari et al., 1989). The subunit composition of NMDA and AMPA receptors follows distinct developmental profiles that determine the functional properties of these receptors (Cull-Candy et al., 2001; Miyamoto et al., 2001). NMDA and AMPA receptors are tetrameric receptors composed of multiple subunits. NMDA receptors are composed of subunits that include the obligatory GluN1 in combination with GluN2 (A-D) and GluN3 (A-B) (Moriyoshi et al., 1991; Das et al., 1998; Hollmann, 1999). The various subunits of the NMDA receptor complex undergo differential maturation during the first weeks of life in rodents, resulting in divergent receptor functions. Brain levels of GluN1 and GluN2A are lowest at birth and peak during the second and third weeks of life (Riva et al., 1994; Zhong et al., 1995; Liu et al., 2004). In contrast, GluN2B subunit levels are high at birth and decrease with age. GluN3A levels are high in newborns and decrease beginning on postnatal day 7 (P7) (Wenzel et al., 1997). AMPA receptors are composed of subunits GluA1, GluA2, GluA3, and GluA4 (Sommer et al., 1991; Hollmann and Heinemann, 1994). AMPA receptor subunit composition is crucial for their conductance, trafficking, and calcium permeability. These properties are primarily dependent on the presence or absence of the GluA2 subunit and its editing (for rev. Henley and Wilkinson, 2016). AMPA receptors either lacking the GluA2 subunit or containing its unedited form are calcium permeable (Lawrence and Trussell, 2000). Expression of GluA1, GluA2, and GluA3 begin to arise during the second week, and GluA4 levels drop after the first week (Zhu et al., 2000). Accurate orchestration of changes in the composition of glutamate receptors during development is critical for synaptic plasticity, the establishment of neuronal circuitry, and normal development of brain function (for rev. Lohmann and Kessels, 2014).

Our previous study demonstrated that BZDs exhibit strong anticonvulsant and anxiolytic effects in immature rats during the first 2 weeks of life. Infantile rats also develop signs of BZD withdrawal after very short exposures (Kubová and Mareš, 1989; Mikulecká et al., 2011; Kubová and Mareš, 2012), suggesting the involvement of neuroadaptive processes in the glutamatergic system.

The present study characterized the effects of short BZD exposure during the first 2 weeks of postnatal life on glutamatergic receptor development in rats. This study investigated whether the previously reported withdrawal phenomena after cessation of BZD exposure in infantile rats were associated with changes in glutamatergic receptors similar to the changes described in mature rats. Further, whether modulation of the GABAergic system by BZDs during the developmental period critical for proper formation of neuronal circuitry resulted in permanent alterations of glutamatergic receptors was also investigated. We used PCR to evaluate alterations in GluN1, GluN2A, GluN2B, and GluN3A subunits of NMDA receptors and GluA1, GluA2, GluA3, and GluA4 subunits of AMPA receptors in the cortex and hippocampus in animals exposed to clonazepam for five consecutive days beginning on P7. We used autoradiography to evaluate binding of the noncompetitive NMDA receptor antagonist MK-801 in several cortical (cingular, frontoparietal, sensorimotor, temporal, piriform, and enthorinal cortices) and hippocampal (the CA1, CA2, and CA3 areas both dorsal and ventral and dentate gyrus of the hippocampus) regions. In addition, binding was also assessed in the substantia nigra, caudate putamen, and the periaqueductal gray. Animals were examined 48 h, 1 week, and 2 months after CZP cessation.

We selected clonazepam (CZP) as our model BZD based on our previous studies demonstrating its profound anticonvulsant and anxiolytic effects in infantile rats (Kubová and Mareš, 1989; Mikulecká et al., 2011) and resulting permanent alteration of cognitive functions and social behavior in exposed animals (Mikulecká et al., 2014a,b).

## Materials and Methods

Experiments were performed in male Wistar albino rats (*n* = 90; 45 controls and 45 CZP-treated). The day of birth was considered day zero (P0). Each litter consisted of 10 males randomly selected at P5 from at least 2 l. Rats were housed in a controlled environment (temperature 22 ± 1°C, humidity 50–60%, lights on 06:00–18:00 h) with free access to food and water. Animals were weaned on P28. All procedures involving animals and their care were performed in accordance with ARRIVE guidelines https://www.nc3rs.org.uk/arrive-guidelines and in compliance with national (Act No 246/1992 Coll.) and international laws and policies (EU Directive 2010/63/EU) for animal experiments and the National Institutes of Health Guide for the Care and Use of Laboratory Animals (NIH Publications No. 8023, revised 1978). The Ethical Committee of the Czech Academy of Sciences approved this experimental protocol (Approval No. 128/2013).

### Pharmacological Treatment

CZP was suspended in physiological saline supplemented with Tween 80 (1 mg/5 ml of saline and one drop of Tween 80) and intraperitoneally injected at 1 mg/kg/day for five consecutive days from P7–P11. At P7, animals in each litter were randomly divided into either control (*n* = 5) or CZP animals (*n* = 5). As previously reported, a single administration of the selected dose of CZP produced anticonvulsant effects for greater than 24 h in immature rats subjected to the PTZ model (Kubová and Mareš, 1989; Mikulecká et al., 2011). Control animals received solvent instead of CZP. Pups were immediately returned to their dams after injection. Separation from the mother during drug administration never exceeded 20 min.

Body weight was monitored daily from P6 to P15. These data were used to calculate relative body weight (body weight at P6 was taken as 100%) to minimize the effects of variability in individual groups. The difference in relative body weights between two consecutive days was used as a measure of weight gain. In P18 and P60 groups, body weight of control and CZP animals was compared prior to euthanasia.

Body temperature of pups was maintained at 32 ± 2°C during CZP administration using an electric heating pad connected to a digital thermometer to compensate for immature thermoregulatory functioning at this age (Conklin and Heggeness, 1971).

Rats were euthanized via decapitation under ether anesthesia 48 h, 1 week, or 2 months after cessation of CZP administration, and brains were rapidly removed and treated as described below.

### Quantitative Real-Time RT–PCR

Hippocampi and sensorimotor cortices were immediately dissected and homogenized in RNAzol RT (Molecular Research Center). Total RNA was extracted using Direct-zol^TM^ RNA MiniPrep (Zymo Research) according to the manufacturer’s instructions. Total RNA (1 μg) was converted to cDNA using the one-step SuperScript^®^ VILO cDNA Synthesis Kit and Master Mix (Invitrogen) according to the manufacturer’s instructions. Samples of cDNA (1 μl) were amplified in 20 μl of PCR reaction mixture containing 5× HOT FIREPol^®^ Probe qPCR Mix Plus (Baria) plus TaqMan probes (Life Technologies; **Table 1**). All qPCR reactions were performed in triplicate in a LightCycler^®^ 480 Instrument (Roche Life Science, Indianapolis, IN, United States) using the following temperature profile: initial denaturation at 95°C for 15 min, followed by 60 cycles consisting of denaturation at 95°C for 18 s and annealing/elongation at 60°C for 60 s. The mean of the crossing point (Cp) obtained from qPCR was normalized to the level of the housekeeping gene Ppia (Cyclophilin A) and used for analysis of relative gene expression by the ΔΔCT method (Livak and Schmittgen, 2001). Briefly, the threshold cycle (Ct) values for the housekeeping gene Ppia generated from PCR reactions were subtracted from Ct values for target genes (NMDARs and AMPAs), yielding ΔCt. The mean ΔCt values of control samples were subtracted from ΔCt values of treated (CZP) samples to yield ΔΔCt. The final value 2–ΔΔCT represents relative fold changes. With this method, we obtained a set of values for each group of control and treated animals. Mean values of the controls were normalized to zero, and values of CZP-treated animals were plotted as percent difference from controls (i.e., zero). For statistics, all values for controls were counted as percent distribution around the mean and compared with treatment groups.

**Table 1:** The list of TaqMan probes used in the study.

Ref. No	Gene symbol	Gene name
Rn00690933_m1	Pipa	peptidylprolyl isomerase A, cyclophilin A
Rn00561341_m1	Grin2a	glutamate receptor, ionotropic N-methyl D-aspartate 2A
Rn00680474_m1	Grin2b	glutamate receptor, ionotropic N-methyl D-aspartate 2B
Rn01436034_m1	Grin1	glutamate receptor, ionotropic N-methyl D-aspartate 1
Rn01448553_m1	Grin3a	glutamate receptor, ionotropic N-methyl D-aspartate 3A
Rn00709588_m1	Gria1	glutamate receptor, ionotropic AMPA 1
Rn01451960_m1	Gria2	glutamate receptor, ionotropic AMPA 2
Rn00583547_m1	Gria3	glutamate receptor, ionotropic, AMPA 3
Rn00568544_m1	Gria4	glutamate receptor, ionotropic, AMPA 4


The reproducibility of assays was evaluated using calculation of the coefficient of variation according to following formula: %CV = σ/μ (σ = SD and μ = mean value). Chosen criterion was % CV < 10.

### Receptor Binding

Brains (five animals per treatment and interval group) were rapidly frozen in pulverized dry ice and stored at -70°C until processing. Brains were sectioned in the coronal plane (20 mm), and serial sections (1-of-5) through the entire brain were thaw-mounted on gelatin-coated slides and stored at -70°C. The set of sections used for MK801 binding assessment contained three microscopic glasses and each glass contained seven to eight brain sections taken from different anatomical regions: the first glass contained sections from levels 3.0 to -0.8 relative to bregma, the second glass from levels -0.8 to -4.5, and the third glass sections from -4.5 to -6.5 relative to bregma (Paxinos and Watson, 1986). Serial and parallel sections were produced from each brain for subsequent autoradiography procedures. Brains of CZP-treated and age-matched control rats were always examined simultaneously.

#### Quantitative Autoradiography

Experiments were performed using previously described procedures (Sakurai et al., 1991). Brain sections were removed from the freezer, dried in a stream of cool air, and immediately washed in 50 mM Tris-acetate buffer (pH 7.4, 23°C) for 30 min to remove endogenous ligands. Sections were incubated in 50-mM Tris-acetate buffer (pH 7.4, 23°C) with 10 μM glycine and 10 μM L-glutamate containing [^3^H]-MK-801 (10 nM; specific activity 17.1 Ci/mmol) in the presence or absence of 10 mM nonlabeled MK-801. Specific binding was calculated as the difference between values obtained from both experimental conditions. Sections were incubated for 2 h at 4°C, washed twice in consecutive buffer solutions, and rinsed with distilled water for 2 s at 4°C. Sections were quickly dried in a mild steam of cold air and arranged in X-ray cassettes with *^3^*H standards (Amersham) followed by 12 weeks exposure to [*^3^*H]-sensitive film (Kodak MR) at 22°C. Each film allowed simultaneous exposure of 21 slides plus one standard, that is, each film included sections from seven animals. Each film contained sections from CZP animals and age matched controls. All slides were processed in one autoradiography assay in order to avoid variability in experimental conditions.

Film was developed at 18–20°C using Kodak D19 developer and fast fixer solutions. In every animal, optical density was assessed as the mean of 10 measurements performed in at least three parallel sections for each evaluated structure. Mean value was calculated and used for statistical evaluation. Optical densities were evaluated using JAVA Jandel image analysis software. A standard curve was generated based on optical density values of the standards, and the specific activity of [*^3^*H]-MK-801 (17.1 Ci/mmol) and tissue thickness (20 μm) were used to express radioactivity values as fmol/mg of protein. Optical density readings of the standards were used to construct a standard curve to determine tissue radioactivity values for accompanying tissue sections (dpm/mm^2^). Tritium standards were previously calibrated to brain homogenates with known protein concentrations to allow transformation of gray values into total binding. Subsequently, dpm/mm^2^ values were converted to fmol/mg protein based on the specific activity of [*^3^*H]-MK-801 (17.1 Ci/mmol) and tissue thickness (20 μm).

### Statistics

Sample size was determined in advance based on previous experience and following the principles of the three Rs (Replacement, Reduction, and Refinement ^1^). All efforts were made to minimize the number of animals used and their suffering. Simple randomization was used to assign each rat to a particular treatment group prior to experimentation. Data analyses were performed by those blinded to treatment. The ages and time points for each group consisted of five to seven animals for the binding study and 10 animals per group used for real-time PCR. Total mRNA subunits are the sum of relative mRNA levels of individual subunits for each time and experimental group plotted as the % of controls. The ratio between GluN2A and GluN2B was calculated from the sum of both subunits in each time and experimental group.

Data were analyzed using GraphPad Prism 7 (GraphPad Software, United States) software. Using the D’Agostino-Pearson normality test, all data sets were first analyzed to determine whether the values were derived from a Gaussian distribution. Outliers were identified with the ROUT test (*Q* = 1%). Differences between age-matched controls and CZP-treated animals were analyzed using two-tailed unpaired *t*-tests, and a *p*-value < 0.05 was required for significance. Data are expressed as the means ± SEM and plotted as % of controls.

## Results

### Effect of CZP Administration on Growth

**FIGURE 1:**
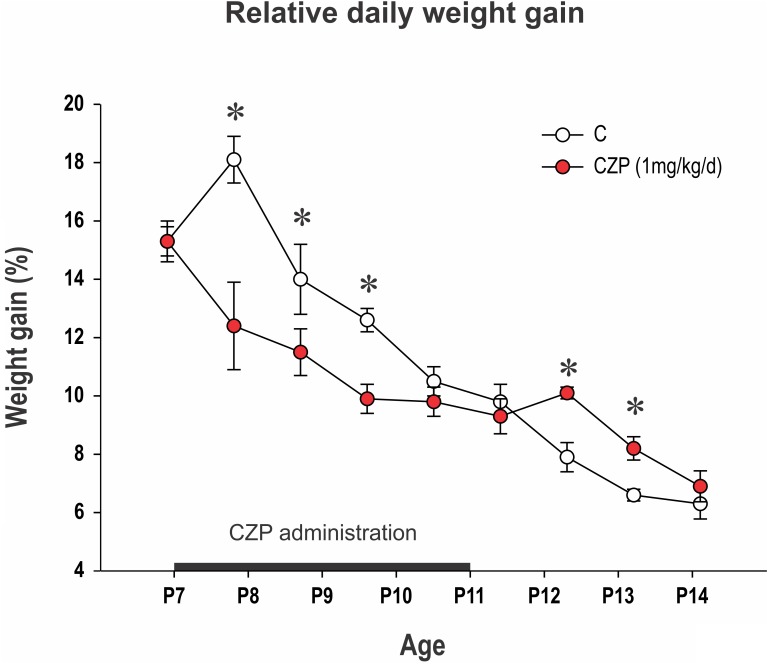
Relative daily weight gain between P7 and P14. Body weight at P6 was normalized to 100% and differences in relative body weight between two consecutive days is presented (*y*-axis). The *x*-axis shows age. CZP (1 mg/kg/day) was administered for five consecutive days beginning at P7 (black line on the *x*-axis). Data are presented as means ± SEM. Controls (empty circles) gained 10.5–18.1% of their body weight daily. CZP animals (red circles) gained significantly less weight up to the fourth day of administration. Asterisks denote significant differences compared to controls. C, controls; CZP, clonazepam-treated animals.

CZP-treated animals gained significantly less weight during the first 4 days of CZP administration compared to controls (**Figure 1**). Animals in the CZP group gained significantly more weight than their littermates after the end of administration (at P12 and P13), compensating for slower growth during CZP administration. There was no difference in body weight between controls and CZP-exposed animals at P18 (45.4 ± 1.1 vs. 44.2 ± 0.7; *p* = 0.3683) or P60 (404 ± 5.5 vs. 391.3 ± 6.6; *p* = 0.2788).

### Effect of CZP Administration on NMDA Receptor Subunit Expression

**FIGURE 2:**
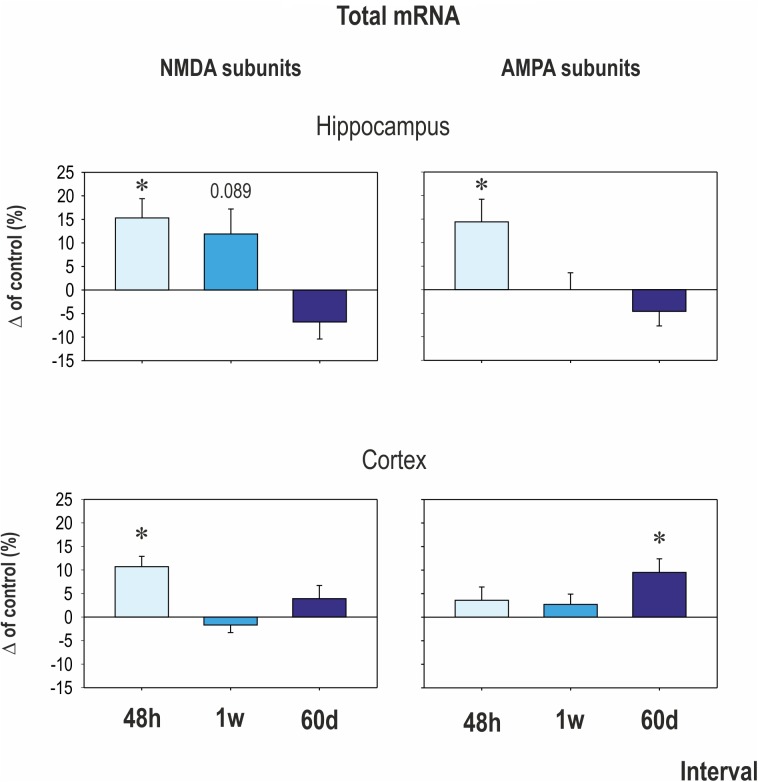
Mean transcriptional levels of evaluated NMDA and AMPA receptor subunits in hippocampus and cortex at three intervals after CZP cessation. mRNA levels were determined using quantitative RT-PCR, and values were converted to a percentage of the control values, which were considered baseline (zero) levels. Mean percent changes in all evaluated subunits are presented (±SEM). Asterisks denote significant differences compared to controls.

**FIGURE 3:**
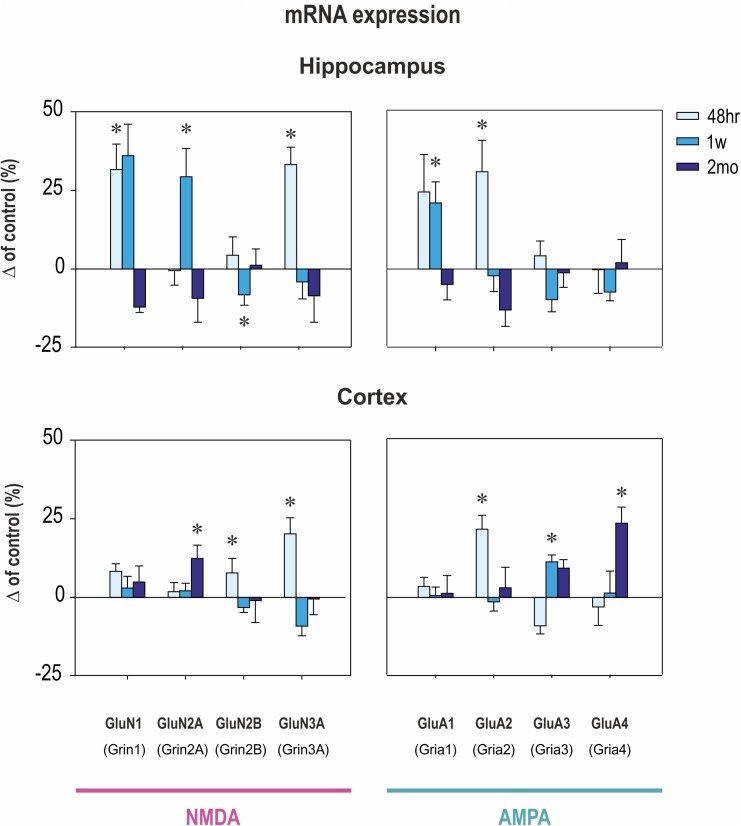
Transcriptional levels of NMDA and AMPA receptor subunits in hippocampus and cortex at three intervals after CZP cessation. mRNA levels were determined using quantitative RT-PCR and values were converted to a percentage of the control values, which were considered as baseline (zero) levels. Data are presented as means ± SEM. Asterisks denote significant differences compared to controls.

Early CZP exposure caused short-term upregulation of GluN1, GluN2A, and GluN3 subunit mRNA in the hippocampus (**Figures 2**, **3**). Total mRNA for all evaluated NMDA subunits remained elevated for 1 week after the end of CZP exposure, and increased expression was significant 48 h after cessation of treatment (*t* = 2.541, df = 72; *p* = 0.0132). Expression of GluN1 and GluN3 was significantly elevated 48 h after CZP cessation (two-tailed unpaired *t*-tests *t* = 2.236, df = 16; *p* = 0.039 and *t* = 2.271, df = 15; 0.0383, respectively). GluN2A subunit mRNA was overexpressed at the 1 week interval (two-tailed unpaired *t*-tests *t* = 2.64; df = 17; *p* = 0.0172). At the same interval, expression of GluN2B was lower in CZP-treated animals compared to controls (*t* = 2.287 df = 16; *p* = 0.0362). This difference in expression was associated with significant shift of the GluN2A/GluN2B ratio (38% in controls and 50% in CZP-exposed animals; *t* = 3.611, df = 20; *p* = 0.0017). Two months after the end of CZP exposure, expression of NMDA subunit mRNA in the hippocampus had normalized and was not significantly different from control levels. In the cortex, total mRNA expression for all evaluated NMDA subunits was elevated 48 h after CZP cessation (*t* = 3.249, df = 72; *p* = 0.0018) mostly due to significant up regulation of GluN3A subunit mRNAs (*t* = 2.198, df = 18; *p* = 0.0413). At later points, expression of mRNA NMDA subunits normalized, with the exception of the GluN2A subunit, which continued to be overexpressed 2 months after the end of CZP exposure (*t* = 3.038, df = 17; *p* = 0.0074) (**Figure 3**).

### Effect of CZP Administration on AMPA Receptor Subunit Expression

In the hippocampus, early exposure to CZP resulted in transiently increased total mRNA at 48 h (*t* = 2.941, df = 74; *p* = 0.0044) mostly due to significant overexpression of the GluA2 subunit (*t* = 2.206, df = 17; *p* = 0.0414) (**Figures 2**, **3**). GluA1 subunit mRNA was overexpressed 1 week after CZP cessation (*t* = 2.844, df = 16; *p* = 0.0117), and expression of both mRNAs normalized, exhibiting no differences at 2 mo. No differences were observed in expression of GluA3 and GluA4 subunit mRNA at any interval examined. In the cortex, GluA2 subunit mRNA was overexpressed at 48 h (*t* = 3.651, df = 18; *p* = 0.0018) and GluA3 subunit mRNA at 1 week (*t* = 3.495, df = 17; *p* = 0.0028) after the end of CZP exposure. Two months after CZP cessation, total mRNA for all AMPA subunits was elevated (*t* = 2.383, df = 76; *p* = 0.0197), primarily due to overexpression of GluA4 subunit mRNA (*t* = 2.553, df = 18; *p* = 0.02).

### Effect of CZP Administration on NMDA Receptor Binding

**FIGURE 4:**
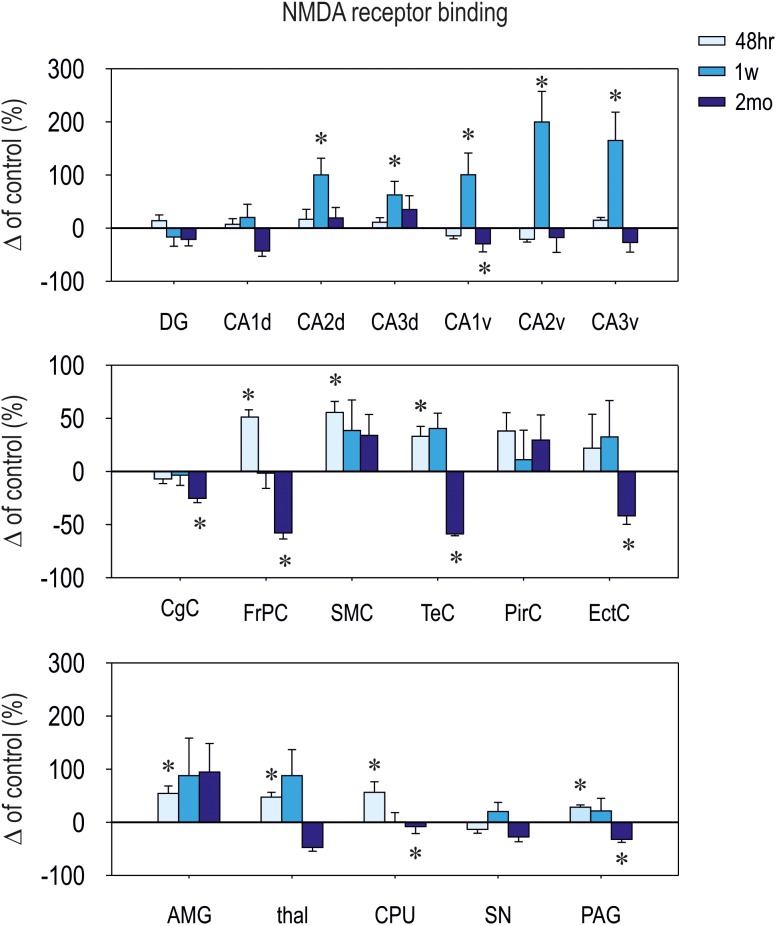
Relative changes in [^3^H]-M-K801 binding to NMDA receptors (means ± SEM). Binding in control animals is considered as baseline (zero) level. On the top: hippocampal structures; in the middle: cortical structures; on the bottom: (amygdala (AMG), thalamus (thal), caudate putamen (CPU), substantia nigra (SN), and periaqueductal gray (PAG). [^3^H]-MK-801 binding was assessed at three different intervals (48 h, 1 week, and 2 months – legend on the top) after CZP cessation. DG, dentate gyrus of the hippocampus; CA, CA1 subfield of the hippocampus (d – dorsal, v – ventral); CA3, CA3 subfield of the hippocampus (d – dorsal, v – ventral); CgC, cingulate cortex; FrPc, frontoparietal cortex; SMC, sensorimotor cortex; TeC, temporal cortex; PirC, piriform cortex; EctC, entorhinal cortex.

**FIGURE 5:**
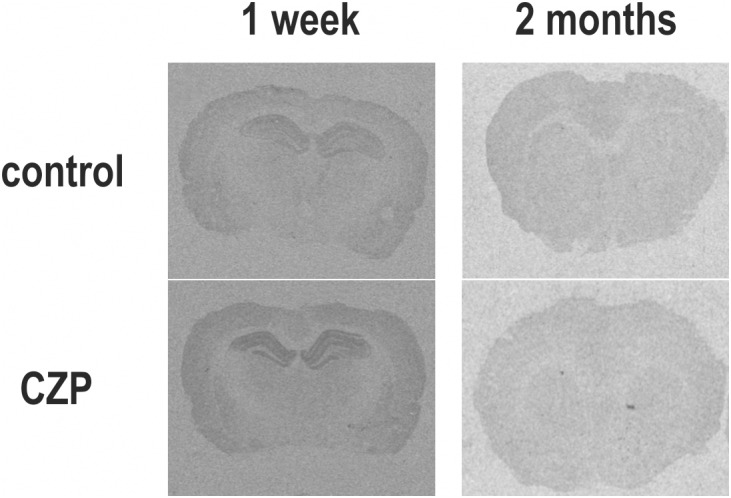
Autoradiograms illustrate the distribution of binding to NMDA receptors labeled with [^3^H]-MK-801 in brain sections from the dorsal hippocampus (–3.3 mm relative to bregma according to Paxinos and Watson, 1986; left panels) and striatum (0.2 mm relative to bregma according to Paxinos and Watson, 1986; right panels) of rats treated with vehicle (C) or clonazepam (CZP), at 1 week (1 w) and 2 months (2 mo) posttreatment. High binding appears as black areas. Compared to controls, animals exposed to CZP exhibited increased [^3^H]-MK-801 binding in the hippocampus 1 week after CZP cessation, whereas 2 months later, binding was lover in the cingulate and frontal cortex and caudate putamen.

Patterns of NMDA ([^3^H] MK-801) receptor binding generally paralleled changes in expression of NMDA receptor subunit mRNAs. Binding was increased within 1 week after CZP cessation in several brain areas (**Table 1** and **Figures 4**, **5**). At 48 h, binding was significantly elevated in several cortical (cx) areas (frontoparietal cx – *t* = 3.011, df = 11; *p* = 0.0119; sensorimotor cx – *t* = 30374, df = 11; *p* = 0.0062 and in the temporal cx – *t* = 2.516, df = 11; *p* = 0.0287), the amygdala (*t* = 2.746, df = 11; *p* = 0.0190), thalamus (*t* = 2.768, df = 11; 0.0183), caudate putamen (*t* = 2.323, df = 11; *p* = 0.0404), and in the periaqueductal gray (*t* = 2.321, df = 11; *p* = 0.0405), whereas at 1 week, increased binding was detected in several areas of the dorsal (CA2 – *t* = 2.700, df = 13; *p* = 0.0182; CA3 – *t* = 2.231, df = 13; *p* = 0.0440) and ventral hippocampus (CA1 – *t* = 2.799, df = 13; *p* = 0.0150; CA2 – *t* = 2.17, df = 13; *p* = 0.0492 and CA3 – *t* = 2.653, df = 13; *p* = 0.0199). Two months after the end of CZP exposure, NMDA receptor binding tended to be lower in almost all evaluated brain regions. Decreases were significant in several cortical areas (cingular cx – *t* = 2.585, df = 11; *p* = 0.0254, frontoparietal cx – *t* = 2.61, df = 11; *p* = 0.0243, temporal cx – *t* = 2.224, df = 11; *p* = 0.0480 and entorhinal cx – *t* = 2.954, df = 11; *p* = 0.0131), the caudate putamen (*t* = 2.444, df = 11; *p* = 0.0326), the CA1 area of the dorsal hippocampus (*t* = 2.777, df = 10; *p* = 0.0195), and in the periaqueductal gray (*t* = 2.298, df = 11; *p* = 0.0422).

[^3^H] MK-801 receptor binding in fmol/mg protein is presented in **Supplementary Table S1**.

## Discussion

Our data demonstrate that exposure to clonazepam during early stages of postnatal development affects NMDA and AMPA receptors both shortly after the end of administration and in the long term. The exact pattern of change depended on the individual receptor subunit, brain structure, and interval after CZP discontinuation. The most pronounced effects were observed within 1 week after CZP cessation in both the hippocampus and cortex. Two days after the end of CZP administration, NMDA receptor subunit mRNAs were elevated in both the hippocampus and cortex, primarily due to GluN3A overexpression. Increased AMPA subunit mRNAs seen in the hippocampus were primarily due to GluA2 mRNA upregulation. The period after abrupt discontinuation of BZD administration is often associated with development of withdrawal symptoms (Michelini et al., 1996; Tobias, 2000, 2005), which present serious treatment complications in both adults and children (Lader, 2014). Despite their clinical importance, withdrawal phenomena, as well as their mechanisms and consequences are only rarely studied in developing animals. We previously reported a rebound increase in seizure susceptibility after both repeated (Kubová and Mareš, 2012) and single (Kubová and Mareš, 1989; Mikulecká et al., 2011) BZD administration in both P7 and P12 rats. These data suggest that the immature brain may be more prone to development of BZD withdrawal phenomena than the adult brain.

Previously published studies on mechanisms underlying dependence and tolerance to benzodiazepines have demonstrated that BZD-induced inhibition is counteracted by modification of the glutamatergic system (for rev. Allison and Pratt, 2003; Korpi et al., 2015) and that chronic administration of BZDs regulates expression of NMDA and AMPA in adult animals. Moreover, juvenile brains responded to BZD exposure with changes in expression of these receptors, but the profile of changes was partially different. Whereas in adult cerebral cortex BZD withdrawal resulted in overexpression of GluN1, GluN2B (Tsuda et al., 1999), and GluA1 (Izzo et al., 2001), in the juvenile cortex expression of GluN2B, GluN3A, GluA2, and GluA3 was upregulated 1 week after CZP cessation. Interestingly, response to early CZP exposure was substantially stronger in the juvenile hippocampus and partially different than in the cortex. Within 1 week after CZP cessation, GluN1, GluN3A, and GluN2A subunits were concomitantly upregulated, whereas GluN2B subunit expression was downregulated. In the hippocampus of adult rats, chronic diazepam resulted in upregulation of both GluN1 and GluN2B subunit mRNAs (Pérez et al., 2003). Overexpression of GluA1 subunit seen in juvenile hippocampus is in agreement with studies in adult rats showing that increased AMPA receptor function in hippocampal CA1 neurons is attributed to the GluA1 subunit (Van Sickle et al., 2004; Xiang and Tietz, 2007; Das et al., 2008). GluA2 subunit was upregulated in the hippocampus of juveniles but not in animals exposed to chronic BZD as adults. Differences in effects of chronic BZD exposure on NMDA and AMPA receptor subunit expression between adult and juvenile animals is difficult to interpret, but most likely, developmental changes in receptor composition and function are playing important roles in these differential molecular responses. Discrepancies in experimental design could also substantially affect results of individual studies.

In line with a study by Tsuda et al. (1998), CZP withdrawal was associated with transient increase of [^3^H]-MK-801binding in most brain areas. Changes in NMDA receptor binding were generally in agreement with upregulated NMDA subunit mRNA expression. Small differences between subunit mRNA and subsequent protein expression might be explained by alterations in transcriptional and translational regulation and possible widespread uncoupling between these two processes (Tebaldi et al., 2012). In addition, changes in subunit composition can substantially affect NMDA binging because NMDA receptors containing GluN3 subunits exhibit reduced sensitivity to MK801 (Chatterton et al., 2002).

While in adult brain, response to drug challenge returns to original baseline, in juvenile rat brain, drug-induced changes may be incorporated as a permanent developmental alteration of the system (for rev. Andersen and Navalta, 2004). In our study, animals were exposed to CZP and subsequent CZP withdrawal during a highly vulnerable period of development. In rodents, the first 4 weeks of life represent a period of growth spurt and increased synaptic plasticity (Dobbing and Smart, 1974; Semple et al., 2013; Lohmann and Kessels, 2014) to create synaptic networks and to properly process environmental stimuli. Synaptic plasticity is particularly driven by chemical neurotransmission with glutamate as a key player (for rev. Lohmann and Kessels, 2014). Glutamatergic pathways undergo striking developmental changes typified by a sequence of changes in composition of both NMDA and AMPA receptors (for rev. Herlenius and Lagercrantz, 2004; Lohmann and Kessels, 2014). Receptor composition determines the function of these receptors and their role in synaptic formation and stabilization (Alvarez et al., 2007). Thus, even transient changes in receptor structure can permanently alter neuronal networks and their functions.

Early in development, expression of GluN1 and GluN2A subunits is low and increases during maturation, whereas GluN2B is highly expressed in the first 2 weeks of life and decreases thereafter (Watanabe et al., 1992; Monyer et al., 1994; Sans et al., 2000). In the hippocampus of CZP exposed animals, expression of GluN1 and GluN2A subunits was upregulated, while GluN2B subunit was concomitantly downregulated, resembling more mature receptor structures. Such a shift may result in reduced ability to undergo synaptic plasticity because GluN2Bs have higher affinity for glutamate (Laurie and Seeburg, 1994) and traffic more rapidly than do GluN2As (Groc et al., 2006). In addition, expression of GluN2A in immature cortex shortens NMDA receptor currents (Flint et al., 1997). Function of NMDA receptors in animals exposed to CZP are further influenced by overexpression of GluN3A that was observed in both the hippocampus and cortex 48 h after the end of treatment. The GluN3A subunit is prominently expressed during a narrow window of intense synaptogenesis during the second and third weeks (Pérez-Otaño et al., 2016). The presence of this subunit decreases Ca^2+^ permeability and sensitivity to Mg^2+^ (Kehoe et al., 2013) and glutamate (Chatterton et al., 2002; Yao and Mayer, 2006). As previously demonstrated, GluN3A mediates some neuroprotective properties, likely by preventing Ca^2+^ influx (Wang et al., 2013). Changes in NMDA receptor subunit expression seen after CZP cessation resemble protective mechanisms and may prevent overexcitation associated with BZD withdrawal and protect immature neurons from damage. On the other hand, exposure to BZDs and consequent changes in receptor structure can alter synaptic plasticity and disrupt synaptic development (Forcelli et al., 2012). GluN3A overexpression has been found to decrease spine density and attenuate LTP induction in the hippocampus (Roberts et al., 2009). In contrast, deletion of this subunit increased spine density (Das et al., 1998). Overexpression of the GluN3A subunit therefore affects formation or elimination of specific synaptic populations that are formed during the second and third weeks of life and modify development of neuronal circuits.

In addition to prominent changes in NMDA receptor subunit expression, CZP exposure resulted in upregulation of GluA2 subunit in both the hippocampus and cortex. Early in development, GluA2 expression is relatively low, and the mRNA remains unedited, even when expressed. AMPA receptors lacking GluA2 or containing unedited GluA2 subunit are calcium permeable. Thus, many AMPA receptors in the immature brain are Ca^2+^ permeable (Kumar et al., 2002; Eybalin et al., 2004; Talos et al., 2006; Brill and Huguenard, 2008).

Early CZP exposure also resulted in long-term alteration of AMPA and NMDA receptors. GluA4 subunit, which is normally tightly developmentally regulated and it is sparse in the adult brain (Zhu et al., 2000), was upregulated in the cortex. Upregulation of GluR4 expression was previously reported in adult animals exposed to morphine and was associated with increased Ca^2+^ permeability (Cabañero et al., 2013). Interestingly, GluA4 containing AMPA receptors mediate fast EPSPs in parvalbumin-containing GABAergic interneurons in the adult brain (Geiger et al., 1997). These neurons play important roles in the regulation of plasticity and learning, and alteration of their function is associated with many neuropsychiatric diseases (for rev. Hu et al., 2014).

At the 2 month interval, [^3^H]-MK-801 binding was decreased in several brain regions, including the ventral hippocampus, caudate-putamen, periaqueductal gray, temporal lobe cortices (cingulate, temporal and entorhinal cortex), and prefrontal cortex. These structures are part of neuronal networks associated with emotional behavior, anxiety, and cognitive functions (for rev. Fyhn et al., 2004; White, 2009; Fanselow and Dong, 2010; Panksepp, 2011; Benarroch, 2012), and both emotional behavior and cognitive function are impaired in animals exposed to BZDs early in life (File, 1986a,b,c, 1987; Schroeder et al., 1997; Mikulecká et al., 2014a,b). What molecular and cellular mechanisms are responsible for these functional alterations and what the exact role NMDA receptors play therein remains to be clarified.

## Conclusion

In conclusion, our study suggests that relatively short exposure to CZP during early postnatal stages of development results in substantial expression changes in NMDA and AMPA receptor subunit mRNAs and [^3^H]-MK-801 binding, both shortly after treatment cessation and over the long term. How these changes affect synaptic plasticity and their impact on these early changes in development of neuronal networks warrants further investigation. We suggest that the changes described in this study may be involved in the development of withdrawal signs after BZD cessation in the immature brain. Together with increased apoptosis (Ikonomidou et al., 2001; Bittigau et al., 2002; Jevtovic-Todorovic et al., 2003) and suppressed neurogenesis (Chen et al., 2009) previously described in immature animals exposed to BZDs, changes in the glutamatergic system participate in behavioral alterations observed later in life.

## Author Contributions

HK, LR, and ZB conceived and designed the experiments. HK, LR, ZB, SM, PM, and DP performed the experiments and analyzed the data. HK and PM wrote the manuscript. All authors reviewed the manuscript.

## Conflict of Interest Statement

The authors declare that the research was conducted in the absence of any commercial or financial relationships that could be construed as a potential conflict of interest.

## Footnotes

1 https://www.nc3rs.org.uk/the-3rs
