# Supplementary material for: Neonatal Clonazepam Administration Induces Long-Lasting Changes in Glutamate Receptors
Source: Front Mol Neurosci. 2018 Oct 11;11:382. doi: 10.3389/fnmol.2018.00382 (PMC6193113; doi:10.3389/fnmol.2018.00382)
Supplement: Supplementary file 1 [file Table_1.DOCX]

**Table 1:** [^3^H]-MK-801binding (fmol/mg protein). Comparison between controls and CZP-exposed animals was done using unpaired two-tailed t-test. Data presented as mean±SEM.

| **Structure** | **Interval 48 hours**  **Mean ±SEM** | | **Interval 1 week**  **Mean ±SEM** | | **Interval 2 months**  **Mean ±SEM** | |
| --- | --- | --- | --- | --- | --- | --- |
|  | **C** | **CZP** | **C** | **CZP** | **C** | **CZP** |
| Cingular cx | 388 ± 25.5 | 361 ± 17.2 | 387.4 ± 15.5 | 373.3 ± 36.7 | 370.2 ± 35.1 | 276.6 ± 15* |
| Frontoparietal cx | 76.1 ± 10.8 | 114.8 ± 5.5* | 74.4 ± 22 | 73.2 ± 10.7 | 97.7 ± 22.5 | 41.3 ± 5.7* |
| Sensorimotor cx | 48.1 ± 5.8 | 74.8 ± 5* | 82.1 ± 22.5 | 113.6 ± 23.7 | 55.8 ± 10.6 | 74.7 ± 11 |
| Temporal cx | 53.4 ± 5.9 | 71 ± 5* | 68.4 ± 15.5 | 96 ± 28.4 | 88.5 ± 25.5 | 36.1 ± 1.4* |
| Piriform cx | 53.4 ± 8 | 73.7 ± 9.2 | 79 ± 30 | 87.7 ± 22 | 44.8 ± 11 | 58 ± 10.6 |
| Entorhinal cx | 67.5 ± 10.9 | 82.2 ± 21.8 | 45 ± 11.5 | 59.6 ± 15.4 | 69.5 ± 14.1 | 40.4 ± 5.6* |
| Amygdala | 41.7 ± 5.6 | 64.3 ± 5.9* | 37.9 ± 8.7 | 71 ± 26.7 | 32.2 ± 3.2 | 62.4 ± 17.3 |
| Caudate putamen | 54.7 ± 9.3 | 80.7 ± 4.9* | 36 ± 7.6 | 43.5 ± 17.6 | 77.2 ± 15 | 40.3 ± 5.4* |
| Thalamus | 46.1 ± 3.8 | 72.2 ± 9.1* | 38.1 ± 8.7 | 38.4 ± 6.7 | 41.2 ± 8.5 | 37.7 ± 5.4 |
| Dentate gyrus | 61 ± 6 | 69.5 ± 6.6 | 51.4 ± 11.3 | 42.9 ± 8.9 | 53.7 ± 12.5 | 42.1 ± 6.5 |
| CA1 dorsal | 93.4 ± 7.6 | 100.8 ± 9.9 | 148.6 ± 32.2 | 178.4 ± 36.9 | 131.4 ± 15.3 | 74.6 ± 13* |
| CA2 dorsal | 76.6 ± 12.3 | 89.2 ± 14.4 | 89.3 ± 15.4 | 172.5 ± 27.1* | 50.2 ± 8.8 | 59.7 ± 9.8 |
| CA3 dorsal | 70.9 ± 10.7 | 78.7 ± 6.1 | 55.6 ± 8.9 | 90.2 ± 12.2* | 43.5 ± 8.4 | 58.7 ± 11.3 |
| CA1 ventral | 75.9 ± 13.5 | 64.8 ± 4.4 | 44.3 ± 11.9 | 88.6 ± 18.1* | 63.7 ± 20.3 | 44.7 ± 9.6 |
| CA2 ventral | 75.9 ± 11.9 | 59.8 ± 4 | 50.3 ± 6 | 134.1 ± 28.9* | 68.3 ± 13.5 | 56.3 ± 19.2 |
| CA3 ventral | 76.3 ± 12.5 | 65 ± 4 | 31.4 ± 4.6 | 83.2 ± 16.7* | 63.2 ± 12.6 | 46 ± 11.5 |
| Substantial nigra | 64.3 ± 12.2 | 55.7 ± 4.7 | 19.9 ± 4 | 23.9 ± 3.4 | 35.5 ± 5.3 | 25.7 ± 3.2 |
| PAG | 47.6 ± 6.1 | 61 ± 2* | 21.1 ± 4.4 | 25.6 ± 5 | 38.2 ± 5.2 | 25.9 ± 2.1* |
